# Why do users stay? Emotional vs. functional attachment in pan-entertainment live streaming platforms

**DOI:** 10.3389/fpsyg.2025.1623568

**Published:** 2025-07-21

**Authors:** Shu Zhang, Tie Ji, Kai Liang, Le Wei, Younghwan Pan

**Affiliations:** ^1^School of Design, Hunan University, Changsha, China; ^2^Faculty of Art and Communication, Kunming University of Science and Technology, Kunming, China; ^3^Department of Smart Experience Design, Kookmin University, Seoul, Republic of Korea; ^4^Anhui Institute of International Business, Hefei, Anhui, China

**Keywords:** pan-entertainment live streaming platforms, continuance intention, cognitive-affective-behavioral intention, attachment theory, PLS-SEM

## Abstract

This study aims to investigate how emotional and functional attachments influence users’ continuance intention on pan-entertainment platforms. With pan-entertainment live-streaming booming, clarifying the psychological forces that keep users loyal is crucial. Prior studies rarely examined how emotional and functional bonds act together. Grounded in the Cognitive-Affective-Behavioral Intention (CABI) framework and attachment theory, this study tests a dual-path model on 306 valid responses, the findings reveal that attraction, perceived enjoyment, interactivity, and entertainment significantly enhance emotional attachment, whereas attraction and interactivity also positively influence functional attachment. Notably, perceived enjoyment and entertainment do not significantly impact functional attachment, suggesting that functional reliance is driven more by instrumental utility than hedonic value. Both emotional and functional attachments are found to be strong predictors of continuance intention. This study advances the CABI framework by incorporating dual-path attachment mechanisms, providing novel insights into user-platform relationships in live streaming contexts. Practically, the findings highlight the importance of real-time interaction features and personalized content recommendations in fostering emotional engagement and strengthening user retention.

## 1 Introduction

Specifically, this study examines how emotional and functional attachment transmit the effects of four cognitive drivers—attraction, perceived enjoyment, interactivity, and entertainment—onto users’ continuance intention. With the rapid proliferation of mobile internet, China’s dominant pan-entertainment live-streaming platforms—Douyin Live, Kuaishou Live, and Huya Live—have evolved into comprehensive venues for gaming, music, talk shows, and other interactive formats. Within these ecosystems, participants can shift effortlessly from audience to broadcaster, erasing the boundary between media outlet and social community. By the end of 2022, China’s online population had reached 1.067 billion, yielding an internet-penetration rate of 75.6% (CNNIC, 2023). As such platforms continue to expand, pinpointing the determinants of users’ continuance intention is essential for sustainable growth and efficient retention strategies (Yang et al., 2025). Retaining an existing user costs roughly one-fifth as much as acquiring a new one, underscoring the economic importance of continuance intention (Hossain and Quaddus, 2012; Bhattacherjee, 2001). Analogous digital-economy pilots—such as China’s Cross-Border E-Commerce Pilot Zones—have demonstrably boosted regional GDP growth, underscoring the macro-economic stakes of platform sustainability (Yang et al., 2024).

Numerous studies have examined the determinants of users’ continuance intention across social media and Pan-entertainment live streaming platforms, leveraging various theoretical approaches. For instance, Expectation-Confirmation Theory (ECT) has been applied to explore how unmet expectations influence ongoing usage decisions (Bhattacherjee, 2001; Yang et al., 2024; Oliver, 1980; Kuo and Hsu, 2022). The Uses and Gratifications Theory has been utilized to understand user motivations and needs in Over-the-Top (OTT) services (Yang, 2022). Meanwhile, factors influencing continued app usage have been examined using the Technology Acceptance Model (TAM) (Beldad and Hegner, 2018), and the persistent use of e-learning platforms has been studied through Self-Determination Theory. Despite these insights, research specifically addressing the unique mechanisms driving user retention on pan-entertainment live streaming platforms remains sparse. Recent work on live-stream classroom platforms also links perceived quality and flow experience to continuance intention, underscoring the need to extend such inquiry to pan-entertainment contexts (Wu and Xie, 2024).

This study aims to bridge this gap by adopting the “Cognitive-Affective-Behavioral Intention” (CABI) framework (Liang and Kee, 2018), a model frequently employed to analyze user behavior across different domains. The CABI framework posits that user actions are shaped by a sequential process involving cognitive, affective, and behavioral elements. Specifically, cognitive factors, such as platform features like interactivity and attraction, influence affective responses, such as emotional and functional attachment, which subsequently determine behavioral outcomes like continuance intention. Prior studies have demonstrated the utility of this framework, such as in assessing passenger satisfaction and behavioral intentions in transportation systems (Gerou, 2022), and exploring user identification with social networking services (Chang and Wu, 2021). However, its application to the context of pan-entertainment live streaming platforms has yet to be fully explored, presenting a unique opportunity for further investigation.

Although numerous studies have identified antecedents of user engagement on social-media platforms (Jia et al., 2023; Zhang et al., 2021; Huang and Zhu, 2016), how specific platform features trigger emotional reactions that translate into behavior remains under-explored. This gap leaves our understanding of the cognitive–affective–behavioral chain incomplete, especially in highly interactive settings such as pan-entertainment live-streaming. Moreover, the forces that attract new users differ from those that sustain continuance; initial adoption is often prompted by external cues like recommendations, whereas ongoing use is anchored in the value and enjoyment users derive directly from the platform (Bhattacherjee, 2001). Clarifying how these distinct drivers feed into continuance intention therefore deserves closer examination.

This research leverages Attachment Theory as a mediating framework within the CABI model to explore how emotional and functional attachments influence the relationship between cognitive factors and behavioral outcomes. Attachment Theory provides an insightful lens to understand the development of emotional bonds and functional dependencies, offering a robust theoretical foundation for analyzing user behavior in interactive digital environments. By integrating these perspectives, this study seeks to uncover the mechanisms underlying user retention and propose actionable strategies for optimizing platform designs to promote sustainable growth. Recent work also shows that stringent environmental regulation can spur technological sophistication in Chinese high-tech manufacturing (Yang L. et al., 2023), illustrating how well-designed governance may simultaneously foster innovation and long-term sustainability in digital service ecosystems.

To guide this investigation, the following research questions are proposed:

*RQ1*: What factors drive users’ continuance intention of pan-entertainment live streaming platforms?

*RQ2*: How and to what extent do these factors influence users’ continuance intention?

This research aims to contribute both theoretically and practically by identifying the key drivers and processes shaping user behavior, offering insights for platform operators to enhance user satisfaction and ensure long-term retention. The theoretical contribution of this study lies in explicitly proposing and empirically validating a dual-path attachment mechanism—emotional and functional—that influences users’ continuance intention on pan-entertainment live streaming platforms. Empirically, this study extends the applicability of the CABI framework to the context of live streaming user behavior. Furthermore, the findings offer direct and practical optimization strategies for enhancing platform operations and user experience design.

Unlike traditional single-vertical services (e.g., Taobao Live for commerce or YouTube Live for gaming), these platforms integrate short-video feeds, algorithmic recommendations, real-time gifting, and influencer incubation into a single mobile ecosystem. Such convergence blurs the boundary between social media and live broadcasting, making them a distinctive research object for continuance-intention studies.

Drawing on 306 valid surveys from Chinese pan-entertainment live-stream users, this study empirically tests the proposed dual-path attachment model and delivers actionable implications for both scholars (theoretical refinement of CABI) and practitioners (user-retention strategies).

## 2 Literature review

### 2.1 Pan-entertainment live streaming platforms

With the widespread adoption of mobile internet and smartphones, pan-entertainment live streaming has become users’ preferred form of interactive entertainment; because revenues from virtual gifts and advertising depend heavily on viewer retention, investigating continuance intention holds considerable commercial and academic significance.

Existing scholarship has mainly interpreted live-streaming behavior through the Technology Acceptance Model (TAM), Expectation-Confirmation Theory (ECT), Uses and Gratifications Theory (U&G), Social Presence Theory, and S-O-R and Flow frameworks. These perspectives, however, remain largely confined to functional appraisal or immersive experience and have yet to clarify how users’ cognitive evaluations of platform features evolve into affective bonds that subsequently drive sustained engagement.

The Cognition–Affect–Behavioral Intention (CABI) framework precisely addresses this gap by delineating a sequential pathway from cognition to affect and then to behavioral intention, a process that aligns closely with the multi-layered participation—from functional use to emotion-driven gifting—observed on pan-entertainment live-streaming platforms.

Nevertheless, prior studies have chiefly focused on user engagement (Hilvert-Bruce et al., 2018), retention (Li et al., 2021), streaming intention (Zhou et al., 2019), gifting behavior (Liu et al., 2022), and commenting motivation (Wang and Li, 2020; Zhang and Pan, 2023), while giving insufficient attention to the emotional resonance elicited by platform functionalities and its impact on continuance intention. Accordingly, the present study adopts the CABI perspective to examine this mechanism systematically, thereby providing empirical evidence to enhance user experience and foster the healthy development of the industry.

### 2.2 Continuance intention

Continuance intention reflects an individual’s internal motivation to repeatedly use a product or service, which, from a consumer behavior perspective, is akin to the decision to repurchase or maintain use (Li, 2021).

In the era of mobile internet, continuance intention has evolved as a multifaceted concept. It now extends beyond simply predicting users’ likelihood to continue using a product (Zhendong and Bingjia, 2022) to examining how platform functionalities facilitate access to desired information and experiences (Tang et al., 2022). This study employs the “Cognition-Affect-Behavioral Intention” theoretical framework to investigate the factors driving continuance intention of pan-entertainment live streaming platforms.

Numerous researchers have explored continuance intention in social media contexts, offering valuable insights. West and Turner categorized user motivations for engaging with social media into cognitive, affective, personal integration, social integration, and tension release factors (West and Turner, 2006). Platforms satisfying these needs are more likely to secure prolonged user engagement. Jeakang Heo observed that a platform’s visual appeal plays a pivotal role in fostering user retention on Pan-entertainment live streaming platforms (Heo et al., 2020). Additional studies identified smoother interactions between users and content creators, as well as features like gifting behaviors, interactive experiences, and personalized audiovisual effects, as significant contributors to user retention (Chen and Lin, 2018; Menon, 2022). Similarly, Singh et al. argued that continuance intention is influenced by platform appeal, entertainment value, and user dependence (Singh et al., 2021). Perceived value has also been identified as a critical factor, with platforms like Facebook benefiting from its role in sustaining engagement (Maqableh et al., 2023). Furthermore, satisfaction and habitual usage have been confirmed as primary drivers of continuance intention with mobile social media apps (Hsiao et al., 2016). In mobile-learning contexts, Hu and Lee verified an expectation-confirmation pathway to continuance intention, suggesting broad applicability of satisfaction mechanisms (He and Li, 2023).

Building on these foundational studies, the present research aims to examine the factors influencing user retention on pan-entertainment live streaming platforms from a distinctive perspective.

## 3 Research hypotheses and model

### 3.1 Attraction

Attraction, initially studied in interpersonal communication, measures the positive evaluations individuals make of others or objects (Walster et al., 1978). In the context of pan-entertainment live streaming platforms, attraction reflects the features and qualities that make a platform appealing and influence users’ engagement. Early studies in e-commerce oversimplified attraction, often treating it as a single-dimensional factor (Campbell et al., 2013; Elbedweihy et al., 2016; Fang, 2014). More recent research has taken a multidimensional approach, such as McCroskey et al.’s framework of task, social, and physical attraction (McCROSKEY et al., 1974). This framework has informed various studies on interpersonal and system-based interactions, emphasizing the role of emotional links in shaping user engagement (Chory, 2013; Duran and Kelly, 1988; McCroskey et al., 2006).

In the field of Information Systems (IS), research suggests that visually appealing and user-friendly designs play a significant role in capturing users’ attention and promoting continuance intention (Cyr et al., 2006; Loiacono et al., 2007). For example, Campbell et al. found that website designs that enhance usability and aesthetics positively influence user loyalty (Campbell et al., 2013; King et al., 2016). Extending to live-stream commerce, Guo et al. showed that a streamer’s physical attractiveness directly shapes consumers’ purchase responses (Allahvirdie Rezaieh et al., 2023). Similarly, Elbedweihy et al. highlighted the role of brand attraction in meeting customer needs and fostering attachment (Elbedweihy et al., 2016).

In the context of pan-entertainment live streaming platforms, this study investigates how platform attraction fosters emotional and functional attachments, which, in turn, enhance users’ continuance intention.

Thus, the following hypotheses are proposed:

*H1*: The stronger the attraction of pan-entertainment live streaming platforms, the stronger the users’ emotional attachment to the platform.

*H2*: The stronger the attraction of pan-entertainment live streaming platforms, the stronger the users’ functional attachment to the platform.

### 3.2 Perceived enjoyment

Perceived enjoyment refers to the intrinsic pleasure users derive from interacting with a platform (Ghani and Deshpande, 1994; Pelet et al., 2017). Today, this interaction between users and technology is more widespread, with users deriving substantial enjoyment not only from social media but also from its intrinsic value in everyday life (Kraut et al., 1998; Walther, 1992). In the context of mobile social networking services (SNS), it encompasses the positive emotions users associate with platform interactions (Gao and Bai, 2014). Turel et al. found that enjoyment derived from social media fosters user dependency on its features (Turel et al., 2011). While Lou et al. emphasized that enjoyment enhances functional attachment by facilitating relationship-building and easy access to information (Lou et al., 2005). The enjoyment users experience on pan-entertainment platforms is critical for fostering emotional bonds and driving engagement. Platforms that prioritize enjoyable experiences can significantly strengthen users’ sense of belonging and functional dependence (Wang C. et al., 2015).

This study examines factors influencing users’ continuance intention of pan-entertainment live streaming platforms. With the rapid evolution of mobile internet, platforms must continuously improve to enhance user enjoyment. This study explores the positive impact of perceived enjoyment on continuance intention, as well as its relationship with emotional and functional attachment.

Therefore, the following hypotheses are proposed:

*H3*: The stronger the perceived enjoyment of pan-entertainment live streaming platforms, the stronger the users’ emotional attachment to the platform.

*H4*: The stronger the perceived enjoyment of pan-entertainment live streaming platforms, the stronger the users’ functional attachment to the platform.

### 3.3 Interactivity

Interactivity refers to the dynamic exchange of actions or communication between two or more individuals, often involving active participation. Scholars have categorized social interactions into simple exchanges, such as polite greetings, and more profound engagements driven by shared interests (Hall, 2018). With the advancement of mobile internet, interactivity has transcended physical boundaries, enabling users to interact seamlessly regardless of distance. The consistency of these interactive patterns fosters user engagement through digital mediums (Çakir, 2015).

In the context of digital platforms, interactivity has been identified as a critical factor influencing user satisfaction and loyalty. For instance, Hilken et al. highlighted how augmented reality (AR) technology enhances interactivity by providing users with immersive and personalized digital experiences, thereby deepening engagement (Hilken et al., 2018). On social media platforms, Chen and Lin observed that interactivity not only simplifies brand communication but also strengthens user-brand relationships through enhanced experiences and feedback mechanisms (Chen and Lin, 2019). Likewise, a streamer’s expertise and entertainment value have been shown to raise viewers’ purchase and follow intentions via trust and flow experience (Jiang et al., 2024). Furthermore, Wang et al. found that younger users are particularly drawn to platforms that facilitate meaningful interactions, reflecting a growing demand for social and functional connections in the digital age (Wang et al., 2019).

For pan-entertainment live streaming platforms, interactivity is embedded in features such as live commenting, virtual gifting, and real-time interactions between users and content creators. These features foster a sense of immediacy and engagement, creating emotional connections and practical dependencies. When users perceive these interactions as meaningful and rewarding, they are more likely to develop both emotional and functional attachments to the platform, which subsequently drive their continuance intention. Therefore, this study proposes the following hypotheses:

*H5*: The stronger the interactivity of pan-entertainment live streaming platforms, the stronger the users’ emotional attachment to the platform.

*H6*: The stronger the interactivity of pan-entertainment live streaming platforms, the stronger the users’ functional attachment to the platform.

### 3.4 Entertainment

Entertainment, a central component of user engagement on pan-entertainment live streaming platforms, is defined as activities that captivate and hold users’ attention, offering satisfaction and emotional relief. Bosshart and Macconi describe entertainment as encompassing pleasure, excitement, relaxation, and diversion (Booshart et al., 1998). Digital platforms have expanded this definition by offering dynamic and personalized entertainment experiences, which cater to users’ diverse preferences and enhance engagement.

Some scholars argue that entertainment is one of the most significant reasons users engage with new media technologies, indirectly influencing their interest in other factors (Hicks et al., 2012; Kaur et al., 2020). In the context of pan-entertainment live streaming platforms, entertainment includes a variety of content such as gaming streams, live music performances, and talk shows. These offerings meet users’ psychological needs for stress relief and satisfaction, encouraging repeated interactions (McQuail, 2010). Previous studies have demonstrated that entertainment satisfaction significantly influences users’ intentions to engage with social media platforms (Tefertiller and Sheehan, 2019; Thussu, 1999). Curras-Perez et al. further identified that entertainment positively affects user attitudes, subsequently influencing both their willingness to recommend the platform and their intent to use it (Curras-Perez et al., 2014). Similarly, Kim and Niehm’s research on websites revealed that entertainment enhances perceived value, which, in turn, strengthens user loyalty to the platform (Kim and Niehm, 2009). Hsu et al. also concluded that a website’s entertainment appeal drives traffic, ultimately affecting customer satisfaction and purchase intentions (Hsu et al., 2012).

The dual influence of entertainment is reflected in its capacity to generate emotional and functional attachment. Engaging content that provides joy and relaxation contributes to emotional attachment by creating positive user experiences. On the other hand, the platform’s ability to deliver reliable and accessible entertainment services enhances functional attachment, as users perceive these features as integral to their daily lives. By addressing both emotional and functional needs, entertainment becomes a pivotal driver of user retention and loyalty. Accordingly, the following hypotheses are proposed:

*H7*: The higher the entertainment value of a pan-entertainment live streaming platform, the stronger the user’s emotional attachment to the platform.

*H8*: The higher the entertainment value of a pan-entertainment live streaming platform, the stronger the user’s functional attachment to the platform.

### 3.5 Attachment theory: emotional and functional attachment

This study employs attachment theory as the foundational framework for investigating the mechanisms driving users’ continuance intentions on pan-entertainment live streaming platforms. Attachment theory originally described enduring emotional bonds between individuals that transcend both time and distance (Ainsworth, 2019; Bowlby, 1982). Although initial studies primarily concentrated on parent–child relationships, subsequent research has expanded the application of attachment theory to various contexts, including social interactions, group dynamics, and technological engagements (Ren et al., 2012). For instance, researchers have demonstrated that attachment bonds between users and brands or content creators significantly influence users’ interactive behaviors on platforms, such as gifting, liking, or sustained viewing (Wan et al., 2017).

Within social media contexts, attachment theory manifests primarily through two dimensions: emotional attachment and functional attachment. These dimensions align closely with the affective and behavioral dimensions of the CABI framework utilized in this study. Specifically, emotional attachment emphasizes the psychological intimacy and enjoyment users experience through platform interactions (Li and Fang, 2019). When pan-entertainment live streaming platforms consistently fulfill users’ emotional needs, such as engaging content and interactive experiences, users are likely to form enduring emotional attachments (Wan et al., 2017). Physiological evidence further links attachment insecurity with lower heart-rate variability and diminished perceived social support (Pourmand et al., 2023). Driven by emotional attachment, users tend to dedicate more time and effort toward maintaining their relationships with the platform (Lu et al., 2022). For example, Slater found that collectors’ continuous collecting behavior fundamentally stems from their strong emotional attachment to the collected items (Slater, 2001). Similarly, pan-entertainment live streaming users who form emotional attachments to platform content or broadcasters are more inclined to remain engaged over time (Wan et al., 2017).

Functional attachment, in contrast, underscores users’ practical dependence on the platform’s specific functionalities (Gan and Li, 2018). When pan-entertainment live streaming platforms effectively satisfy users’ practical needs such as information access, real-time interaction, and personalized content recommendations, users develop functional dependencies on the platform (Xiang et al., 2022). For instance, YouTube effectively enhances users’ functional attachment and significantly increases their long-term loyalty and continuance intentions by offering personalized content recommendations, multi-device compatibility, and user-friendly features (Lee and Lehto, 2013). Compared to emotional attachment, functional attachment represents an instrumental value, indicating users’ higher reliance on practical and usability features, further promoting sustained usage intentions (Gan and Li, 2018). Comparable patterns appear in mobile-phone dependence, where adult attachment predicts functional reliance via loneliness (Ho et al., 2024).

Conversely, maladaptive attachment styles can fuel excessive shopping through defensive splitting, highlighting the dark side of functional attachment (Yang X. et al., 2023).

Together, these findings suggest that both emotional and functional attachments play distinct yet complementary roles in influencing users’ continued engagement with pan-entertainment live streaming platforms. Based on this theoretical foundation, the following hypotheses are proposed:

*H9*: The stronger the emotional attachment users have to pan-entertainment live streaming platforms, the stronger their intention to continue using the platform.

*H10*: The stronger the functional attachment users have to pan-entertainment live streaming platforms, the stronger their intention to continue using the platform.

### 3.6 Research model

**Figure 1:**
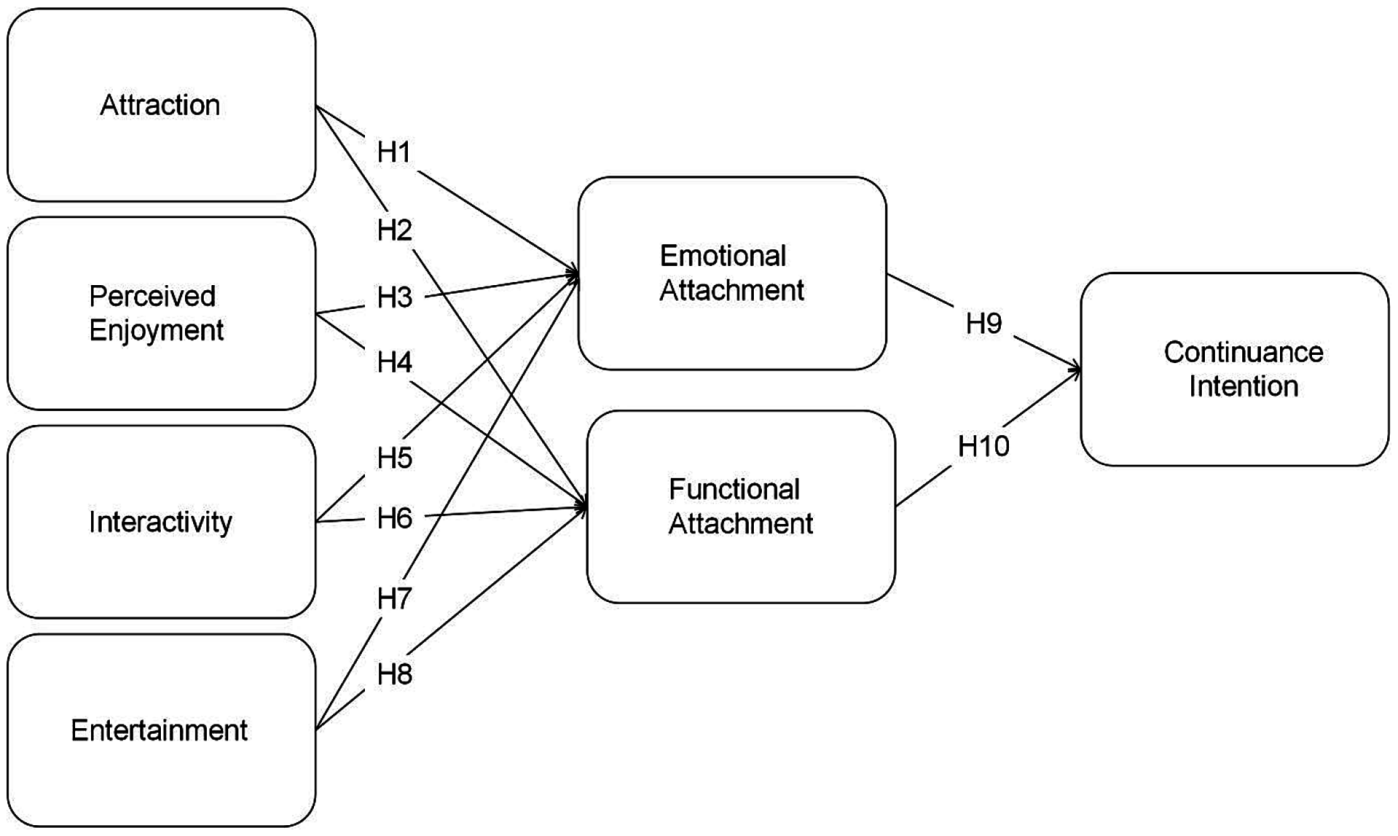
Predictive model of factors influencing continuance intention for pan-entertainment live streaming platforms.

Based on these hypotheses, a research model incorporating emotional and functional attachment is proposed to explore users’ continuance intention for pan-entertainment live streaming platforms. The model is grounded within the “cognition-affect-behavioral intention” framework, as illustrated in Figure 1.

## 4 Methods

### 4.1 Questionnaire design

This study employed a multi-item scale to measure each variable, as it offers greater reliability and reduces measurement errors compared to single-item scales. To ensure content consistency and construct validity, the items for each variable were adapted from prior studies with well-established psychometric properties. These scales, having undergone extensive testing in previous research, demonstrated robust reliability and validity. The original items were revised and contextualized to suit the domain of pan-entertainment live streaming platforms. In total, the questionnaire consisted of 28 items, distributed across seven latent variables, with each variable measured by four items. The survey was organized into two main sections: the first section collected respondents’ demographic information, including gender, age, education level, and usage frequency of pan-entertainment live streaming platforms or similar applications; the second section assessed the latent constructs related to the theoretical model of users’ continuance intention. All items were rated using a five-point Likert scale, where 1 indicated “strongly disagree,” 3 indicated “neutral,” and 5 signified “strongly agree.” Table 1 presents an overview of the measured variables, the number of items per construct, representative item examples, and corresponding literature sources.

**Table 1:** Measurement items and sources for constructs in the CABI model of pan-entertainment live streaming.

Variables	Number of items	Sample of items	Sources reference
Attraction (A)	4	I think the interface of the pan-entertainment live streaming platform has beautiful icon ideas and comfortable font size	Heo et al. (2020) and Permadani and Hartono (2022)
Perceived enjoyment (PE)	4	I like to use the Pan-Entertainment Live Streaming Platform	Chen and Lin (2018) and Nguyen (2015)
Interactivity (I)	4	I often interact with people on live pan-entertainment platforms	Pang et al. (2020) and Yang et al. (2022)
Entertainment (E)	4	Exciting program content for a pan-entertainment live streaming platform	Chen and Lin (2018), Luo (2002), and Sharabati et al. (2022)
Emotional attachment (EA)	4	I’d like to be friends with other users of the PanEntertainment Live platform	Xu (2023) and Zhu et al. (2020)
Functional attachment (FA)	4	In order to accomplish my experience or other goals, pan-entertainment live streaming platforms can provide data resources	Xiang et al. (2022) and Zhang et al. (2022)
Continuance intention (CI)	4	The pan-entertainment live streaming platform is worth my continued viewing pleasure	Loh et al. (2022) and Elsotouhy et al. (2024)

The constructs are measured using a five-point Likert scale, ranging from 1 (representing ‘strongly agree’) to 5 (defining ‘strongly disagree’).

The items measuring attraction were adapted from Heo et al. (2020) and Permadani and Hartono (2022). Perceived enjoyment items were drawn from Chen and Lin (2018) and Nguyen (2015). Interactivity was assessed using established scales developed by Pang et al. (2020), Nguyen (2015), Yang et al. (2022), and Wang et al. (2023). Items capturing entertainment were adapted from Chen and Lin (2018), Luo (2002), Sharabati et al. (2022). The emotional attachment scale was derived from Xu (2023) and Zhu et al. (2020), while functional attachment items were based on Zhang et al. (2022) and Xiang et al. (2022). Lastly, continuance intention was measured using items developed by Loh et al. (2022) and Elsotouhy et al. (2024).

To enhance content validity, a pilot survey was conducted among experienced users of short video social media platforms prior to the questionnaire’s official distribution. Participants were invited to identify items they perceived as “unclear,” “ambiguous,” or “insufficiently differentiated.” Based on their feedback, appropriate modifications were made, including the revision, addition, or removal of certain items. These refinements yielded a finalized measurement scale specifically tailored to assess the determinants of continuance intention within the context of pan-entertainment live streaming. Furthermore, exploratory factor analysis confirmed that Perceived Enjoyment and Entertainment loaded onto distinct factors (Δχ^2^ = 54.27, *p* < 0.001), thereby addressing potential multicollinearity concerns.

### 4.2 Sample characteristics

All 306 respondents were Chinese users of pan-entertainment live-streaming platforms. Their age, income and education profiles closely match national internet statistics (CNNIC, 2023). This alignment strengthens the external validity of our findings. Users within the Chinese market present unique characteristics in terms of age distribution, income structures, educational backgrounds, and occupational diversity. This detailed demographic profiling allows for a deeper understanding of behavioral patterns and psychological mechanisms on these platforms, providing a solid foundation for future theoretical and practical research within the context of the Chinese market. After applying these data screening criteria, the final sample consisted of 306 valid responses. The demographic characteristics of these respondents are summarized below. The survey introduction highlighted the significance of participants’ contributions in advancing research on pan-entertainment mobile live streaming platforms. Participants were informed that their responses would be used exclusively for academic purposes and would remain confidential, with no personally identifiable information collected. To ensure anonymity and build trust, assurances were given that the data would not be shared with third parties. To encourage thoughtful and genuine responses, an incentive system was introduced. Each participant who completed the survey received a 10 RMB WeChat red envelope as a token of appreciation. This reward was chosen for its simplicity and accessibility, making it convenient for participants to redeem. The incentive aimed to acknowledge participants’ time and effort while promoting honest and accurate responses to the survey questions.

To ensure data quality and enhance participation rates, several measures were implemented. The study utilized an online survey platform, Wenjuanxing—a widely used professional tool for conducting surveys in China—to collect data between July and October 2022. A total of 317 questionnaires were initially gathered. After excluding 11 invalid responses, 306 valid questionnaires were retained.

The screening process applied two main criteria to ensure data validity. First, respondents were required to have prior experience using mobile pan-entertainment live streaming platforms; eight responses from individuals who had never used such platforms were excluded. Second, the quality of responses was evaluated based on the time taken to complete the questionnaire and the variation in answers. Submissions with identical or nearly identical responses throughout, or those completed in under 15 s, were deemed invalid and removed. In total, 15 questionnaires were excluded based on these criteria.

As shown in the table below, 50.33% of the sample were male, and 49.67% were female. In terms of age distribution, the majority of respondents (50.00%) were “over 30 years old,” followed by 43.46% aged 20–30 years. Regarding income, 42.48% reported earning “over 3,000 RMB,” while 39.87% earned between 1,000 and 3,000 RMB. In terms of education level, the largest group (34.64%) had a “bachelor’s degree.” Regarding occupation, the majority of respondents (70.26%) were “employees in enterprises or public institutions.” Lastly, in terms of time spent on the platform, over 30% of respondents reported spending “1–2 h” on pan-entertainment live streaming platforms, while 32.03% spent “30 min to 1 h.” According to the 51st Statistical Report on China’s Internet Development (CNNIC, 2023), the national internet population is composed of 51% males and 49% females, with the largest age clusters falling between 20–39 years (38.1%) and 40–49 years (19.9%). In our sample, males account for 50.33% and females 49.67%, closely mirroring the national gender split. Likewise, respondents aged 20–30 years (43.46%) and over 30 years (50.00%) align with the dominant 20–49 age brackets reported by CNNIC. Income and education levels also converge on the urban middle-income cohort that drives most live-stream consumption in China. These parallels indicate that the 306 valid cases reasonably represent the broader Chinese netizen profile, thereby supporting the external validity of the study’s findings (CNNIC, 2023) (Table 2).

**Table 2:** Descriptive statistics of demographic variables (*N* = 306).

	Option	Frequency	Percentage (%)	Cumulative percentage (%)
Gender	Male	154	50.327	50.327
	Female	152	49.673	100
Age	Under 20 Years	20	6.536	6.536
	20–30 Years	133	43.464	50
	Above 30 Years	153	50	100
Income	Below 1,000 RMB	54	17.647	17.647
	1,000–3,000 RMB	122	39.869	57.516
	Above 3,000 RMB	130	42.484	100
Educational level	High School or Below	56	18.301	18.301
	Associate Degree	74	24.183	42.484
	Bachelor’s Degree	106	34.641	77.124
	Graduate Degree or Higher	70	22.876	100
Occupation	Student	16	5.229	5.229
	Employee in Enterprises or Institutions	215	70.261	75.49
	Freelancer	55	17.974	93.464
	Retired	20	6.536	100
Time spent on general entertainment live streaming platforms	Less than 30 Minutes	18	5.882	5.882
	30 Minutes to 1 Hour	98	32.026	37.908
	1 to 2 Hours	115	37.582	75.49
	More than 3 Hours	75	24.51	100
Total		306	100	100

## 5 Results

### 5.1 Measurement model evaluation

#### 5.1.1 Reliability and validity assessment

Internal consistency reliability is a statistical technique used to evaluate the stability and trustworthiness of measurements within a study. It helps identify the degree of measurement error, ensuring that the test results are consistent and reliable. Two widely used methods for assessing internal consistency are Cronbach’s Alpha (CA) and Composite Reliability (CR). In this study, Cronbach’s Alpha was employed to evaluate the questionnaire’s reliability during the pre-test phase. The *α* coefficient, which ranges from 0 to 1, indicates acceptable reliability when it exceeds 0.7 and is considered very high above 0.9. Composite Reliability, on the other hand, measures the internal consistency of structural indicators, with a threshold of 0.7 generally accepted, though values as low as 0.6 are also deemed sufficient (Hair, 2009; Fornell and Larcker, 1981). Factor loading analysis assesses the correlation between individual variables and their respective factors, with loadings ranging from −1 to 1. The squared loading values represent the proportion of variance explained by a factor. A threshold of 0.7 is typically used, and values above this indicate acceptable factor loadings (Hair et al., 2014). Another method applied in this study was Average Variance Extracted (AVE), which evaluates the proportion of variance in observed variables attributable to the latent construct. AVE is commonly used to test both reliability and discriminant validity, with values above 0.5 being satisfactory (Hair et al., 2014). The detailed results of the reliability and validity analysis conducted in this study are summarized in Table 3.

**Table 3:** Reliability and validity analysis.

		Factor loading	*T*-statistic	*P*-value	CA	CR	AVE
A	A1 < - A	0.888	75.351	0.000	0.860	0.905	0.839
	A2 < - A	0.814	35.623	0.000			
	A3 < - A	0.814	37.306	0.000			
	A4 < -A	0.839	47.189	0.000			
PE	PE1 < -PE	0.817	37.020	0.000	0.857	0.903	0.836
	PE2 < - PE	0.850	51.096	0.000			
	PE3 < -PE	0.847	52.497	0.000			
	PE4 < -PE	0.831	38.794	0.000			
I	I1 < -I	0.845	49.287	0.000	0.867	0.909	0.845
	I2 < -I	0.816	35.154	0.000			
	I3 < -I	0.888	67.702	0.000			
	I4 < -I	0.831	38.911	0.000			
E	E1 < -E	0.786	30.010	0.000	0.847	0.897	0.828
	E2 < -E	0.844	48.980	0.000			
	E3 < -E	0.842	43.058	0.000			
	E4 < -E	0.840	46.649	0.000			
EA	EA1 < -EA	0.870	64.089	0.000	0.863	0.907	0.842
	EA2 < -EA	0.834	44.115	0.000			
	EA3 < -EA	0.875	71.925	0.000			
	EA4 < -EA	0.787	40.973	0.000			
FA	FA1 < - FA	0.804	38.611	0.000	0.837	0.891	0.819
	FA2 < - FA	0.821	40.975	0.000			
	FA3 < - FA	0.811	44.064	0.000			
	FA4 < -FA	0.842	47.861	0.000			
CI	CI1 < -CI	0.796	34.865	0.000	0.818	0.880	0.804
	CI2 < -CI	0.803	33.722	0.000			
	CI3 < -CI	0.819	42.433	0.000			
	CI4 < -CI	0.799	38.897	0.000			

#### 5.1.2 Discriminant validity

Discriminant validity analysis ensures that statistically significant distinctions exist between different constructs. For discriminant validity to be established, items belonging to separate constructs should not exhibit high correlations. A correlation above 0.85 suggests that the constructs might overlap excessively, indicating they measure the same underlying concept. To verify discriminant validity, this study uses the more stringent AVE method. According to Fornell and Larcker, the square root of the AVE for a construct should exceed the correlation coefficients between that construct and other variables. This ensures that factors are distinct from one another. The diagonal values in the matrix represent the square root of the AVE for each factor, which must be greater than the off-diagonal standardized correlation coefficients. The correlation coefficients are presented in the lower triangle of the matrix. The findings confirm discriminant validity, and the detailed results are displayed in Table 4.

**Table 4:** Discriminant validity.

	**A**	**PE**	**I**	**E**	**EA**	**FA**	**CI**
A	**0.839**						
PE	0.232	**0.836**					
I	0.346	0.343	**0.845**				
E	0.217	0.215	0.301	**0.828**			
EA	0.495	0.431	0.511	0.554	**0.842**		
FA	0.321	0.279	0.466	0.264	0.548	**0.819**	
CI	0.466	0.373	0.477	0.321	0.628	0.579	**0.804**

Bold diagonal elements represent the square root of AVE; off-diagonal elements are latent-variable correlations.

Subsequently, the Heterotrait-Monotrait Ratio (HTMT) was utilized, which represents the ratio of the average correlations between different traits (between-trait) to the average correlations within the same trait (within-trait). This ratio indicates the degree of discriminant validity between different constructs. As shown in the table below, all HTMT values between pairs of variables in this study are below 0.85, demonstrating good discriminant validity among the variables (Table 5).

**Table 5:** HTMT discriminant validity.

	A	PE	I	E	EA	FA	CI
A							
PE	0.265						
I	0.399	0.398					
E	0.251	0.252	0.352				
EA	0.571	0.501	0.591	0.646			
FA	0.378	0.327	0.546	0.313	0.644		
CI	0.554	0.439	0.566	0.385	0.747	0.698	

### 5.2 Structural equation modeling

#### 5.2.1 Model fit and *R*^2^

The *R*^2^ value of endogenous latent variables is generally interpreted as follows: an R^2^ above 0.67 suggests strong explanatory power, values between 0.33 and 0.67 indicate moderate explanatory power, values from 0.19 to 0.33 reflect weak explanatory power, and values below 0.19 imply minimal explanatory power. In this study, the results are summarized in the table below. For emotional attachment (E), the *R*^2^ is 0.557, with an adjusted *R*^2^ of 0.551. This indicates that the model accounts for 55.1% of the variance in emotional attachment, representing strong explanatory power. For functional attachment (F), the *R*^2^ is 0.267, and the adjusted *R*^2^ is 0.257, meaning the model explains 25.7% of the variance in functional attachment, which corresponds to moderate explanatory power. Lastly, for continuance intention (G), the *R*^2^ is 0.473, with an adjusted *R*^2^ of 0.469, showing that the model accounts for 47.3% of the variance in continuance intention, also reflecting strong explanatory power (Tables 6, 7).

**Table 6:** Model fit and *R*^2^.

	***R*** ^**2**^	**Adjusted *R*** ^**2**^
EA	0.557	0.551
FA	0.267	0.257
CI	0.473	0.469

**Table 7:** PLS model fit indices.

Fit index	Recommended	Study value
SRMR	<0.08	0.049
d_ULS	n.s.	0.716
d_G	n.s.	0.354
NFI	>0.90	0.925
RMS_theta	<0.12	0.085

SRMR = Standardized Root Mean Square Residual; NFI = Normed Fit Index; RMS_theta = Root Mean Square Theta; d_ULS and d_G have no universal cut-off and are compared against their bootstrap distributions.

#### 5.2.2 Direct and indirect effects (bootstrapping, 5,000 resamples)

**Figure 2:**
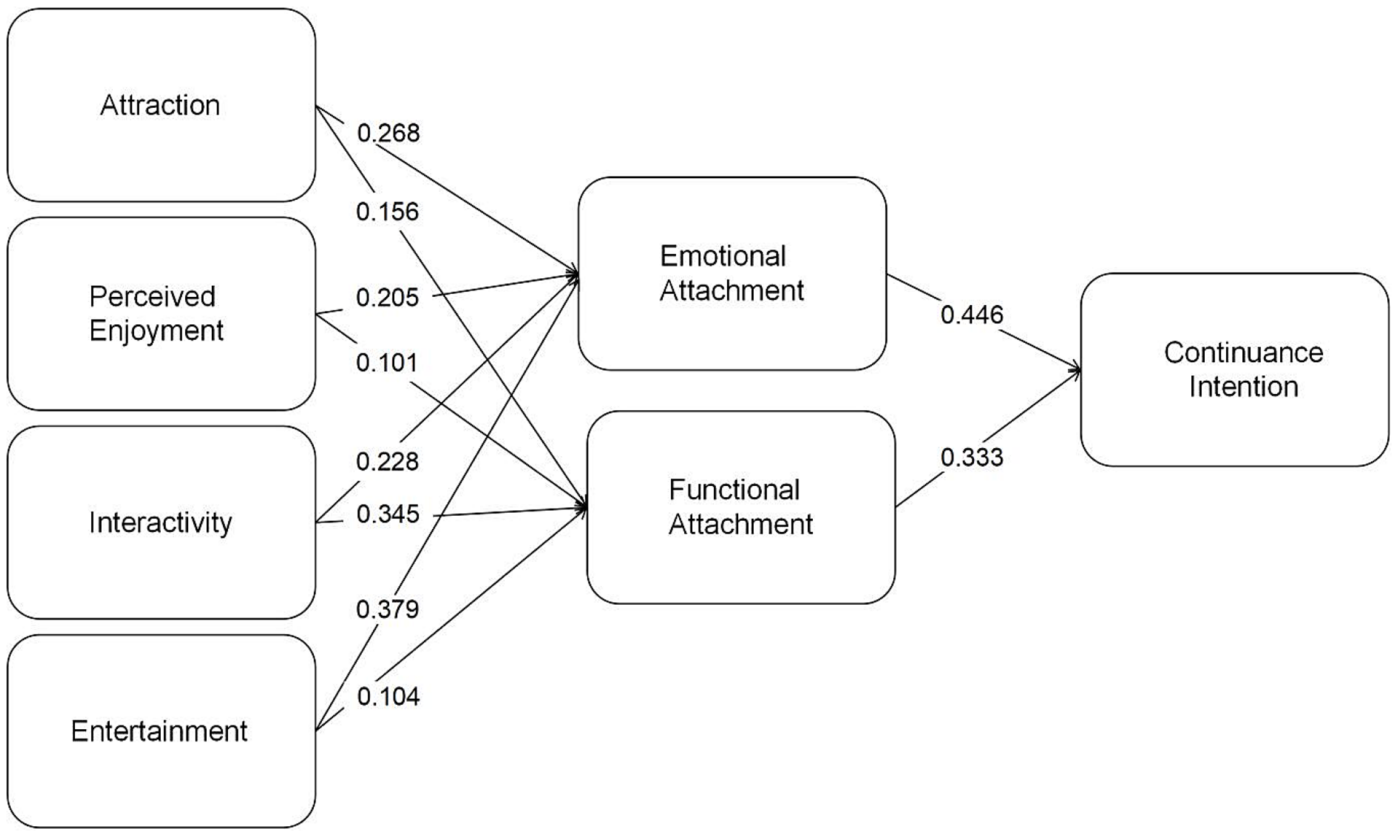
Structural model results with standardized path coefficients.

Bias-corrected percentile bootstrap (5,000 resamples) was employed; indirect-path *β*, 95% CI, and VAF are reported below. The relationships between the hypotheses in this study were assessed using the magnitude and significance of path coefficients. Path coefficients, calculated after standardizing the sample data, range from −1 to 1. A coefficient closer to 1 signifies a stronger positive correlation, while one closer to −1 indicates a stronger negative correlation. To determine significance, the *T*-value is obtained by dividing the path coefficient by its standard deviation. Based on prior research, when the sample size exceeds 30, the normal distribution’s quartiles can be used as critical thresholds. A *T*-value exceeding these thresholds indicates statistical significance at specific error levels. Commonly applied critical values are 1.96 for a 5% significance level, 2.57 for a 1% significance level, and 3.29 for a 0.1% significance level (Hair et al., 2014). This study utilized the bootstrapping method to compute path coefficients and T-values, with 5,000 bootstrap samples. The structural model’s path coefficients are visualized in Tables 8, 9, and the detailed results are displayed in the accompanying table (Figure 2).

**Table 8:** Path coefficients of the PLS structural equation model.

	Initial sample	Sample mean	STDEV	*T*-statistic	*p*-value
A - > EA	0.287	0.288	0.042	6.836	0.000
A - > FA	0.154	0.157	0.052	2.969	0.003
PE - > EA	0.204	0.206	0.043	4.727	0.000
PE - > FA	0.102	0.102	0.056	1.832	0.067
I - > EA	0.228	0.228	0.044	5.129	0.000
I - > FA	0.347	0.348	0.053	6.594	0.000
E- > EA	0.379	0.378	0.039	9.646	0.000
E - > FA	0.104	0.103	0.057	1.823	0.068
EA - > CI	0.445	0.444	0.047	9.410	0.000
FA - > CI	0.335	0.337	0.050	6.685	0.000

H1–H3, H5–H7, H9, and H10 were supported, whereas H4 and H8 (PE → FA, ENT → FA) were not significant (*p* > 0.05).

**Table 9:** Indirect effects and significance levels in the structural model.

	Initial sample	Sample mean	STDEV	*T*-statistic	*p*-value	2.50%	97.50%
A - > EA- > CI	0.128	0.128	0.023	5.431	0.000	0.085	0.177
FA - > EA- > CI	0.091	0.092	0.023	4.031	0.000	0.050	0.138
I - > EA- > FA	0.101	0.101	0.024	4.279	0.000	0.058	0.149
E - > EA- > CI	0.168	0.168	0.022	7.493	0.000	0.125	0.213
A- > FA- > CI	0.052	0.053	0.020	2.613	0.009	0.017	0.094
PE - > FA- > CI	0.034	0.035	0.020	1.682	0.093	−0.002	0.079
I - > FA- > CI	0.116	0.117	0.025	4.645	0.000	0.072	0.170
E - > FA- > CI	0.035	0.035	0.020	1.747	0.081	−0.003	0.076

## 6 Discussion

### 6.1 Key findings

Grounded in the “Cognitive-Affective-Behavioral Intention” framework, this study developed a model to examine the factors influencing users’ continuance intention of general Pan-entertainment live streaming platforms, focusing on emotional and functional attachment. The hypothesis testing and analysis yielded the following key findings:

Firstly, prior studies have shown that functional features play a crucial role in attracting and retaining user engagement on social media platforms. For instance, Chang et al. demonstrated that social network services can enhance users’ emotional connection and identification when they effectively meet user needs (Chang and Wu, 2021). Similarly, Maqableh et al. highlighted how social, personal, and hedonic value significantly contribute to users’ continuance intention with platforms like Facebook (Maqableh et al., 2023). West and Turner also found that users are more likely to maintain their platform usage when their cognitive, emotional, and social needs are satisfied (West and Turner, 2006). Meeting those four expectations thus translates into stronger continuance intention.

Secondly, emotional and functional attachments emerged as critical psychological constructs that enhance user satisfaction and engagement. Emotional attachment reflects users’ affection and attraction toward a platform, while functional attachment represents their reliance on the platform’s features to fulfill specific needs (Wan et al., 2017). When platform features consistently meet user needs, they foster emotional bonds, prompting users to invest more time and effort (Choi, 2013; Xianjin et al., 2017). Lou and other researchers have also emphasized that platforms facilitating interaction and relationship-building foster functional attachment, further reinforcing continuance intention behavior (Lou et al., 2005).

Finally, this study reveals the mechanisms through which cognitive and emotional dimensions influence users’ continuance intention. The findings indicate that attraction, perceived enjoyment, interactivity, and entertainment significantly contribute to emotional attachment, while attraction and interactivity also play a role in fostering functional attachment. However, perceived enjoyment and entertainment did not show a significant effect on functional attachment, which aligns with the findings of Çakir et al., who suggested that social interactions on digital platforms primarily reinforce emotional bonds, thereby promoting sustained engagement (Wan et al., 2017; Çakir, 2015). Attachment anxiety has also been shown to heighten vulnerability to cyber-bullying when self-disclosure increases, revealing another emotional cost of social media use (Wang and Xuan, 2024). A possible explanation for this non-significant effect is that users may prioritize a platform’s instrumental utility over its hedonic attributes when developing functional reliance. This suggests that functional attachment is more likely driven by the platform’s efficiency in facilitating information access, optimizing interaction quality, and ensuring service reliability rather than by its entertainment-oriented attributes.

Compared to Chen and Lin (2018), who emphasized social interaction as key to continuance intention, this study explicitly examines the unique contributions of emotional and functional attachments to continuance intention. Unlike Singh et al. (2021), which focused primarily on addiction factors, our findings highlight the complementary roles of practical utility and emotional experience in sustained user behavior. A recent serial-mediation model on social-media addiction likewise underscores how affect and FoMO interlock with practical motives to prolong engagement (Zhang et al., 2023).

From a practical standpoint, platform operators can enhance user retention by refining real-time interaction mechanisms and improving personalized content recommendations, thereby reinforcing users’ dependence on the platform’s functional aspects. Evidence from live-stream e-commerce further indicates that affordances such as vivid product display markedly raise purchase intention (Tang et al., 2024). Similarly, Elbedweihy et al. found that improving functional features strengthens functional attachment, which, in turn, drives continuous usage (Elbedweihy et al., 2016).

Notably, this study found that while entertainment and perceived enjoyment significantly influenced emotional attachment, they did not significantly impact functional attachment. This suggests that when developing functional dependencies, users tend to prioritize the platform’s instrumental utility—such as service efficiency, information access, and interface reliability—over its hedonic or entertainment-oriented features. Consequently, platform operators should place greater emphasis on usability and operational convenience in their user retention strategies, rather than relying solely on entertainment value.

### 6.2 Theoretical implications

This study advances theoretical understanding in the field of user retention on Pan-entertainment live streaming platforms by integrating attachment theory into the Cognitive-Affective-Behavioral Intention (CABI) framework, addressing key gaps in prior research. While much of the existing literature on continuance intention has primarily examined cognitive factors such as satisfaction and perceived value or focused on isolated affective responses (Bhattacherjee, 2001; Hsiao et al., 2016), this research takes a different approach by incorporating emotional and functional attachment as dual mediators. This integration offers a more comprehensive perspective on how users’ psychological bonds and functional dependencies shape behavioral intentions. Rather than treating cognition and behavior as a direct sequence, this study emphasizes the dynamic interplay between affective and functional dimensions in interactive digital environments, expanding the explanatory power of the CABI framework (Liang and Kee, 2018).

In applying attachment theory to Pan-entertainment live streaming platforms, this research also identifies mechanisms that set these platforms apart from static social media (Wan et al., 2017) or e-commerce environments (Lu et al., 2022). Unlike traditional platforms, real-time interactivity (e.g., live commenting and virtual gifting) and entertainment-driven engagement strengthen not only emotional attachment (by fostering parasocial relationships with content creators) but also functional attachment (by enhancing platform reliability and usability). This dual-path mediation challenges the conventional assumption that functional dependency is primarily driven by utilitarian value (Gan and Li, 2018), highlighting the role of emotional resonance as a complementary driver of continued use in highly interactive digital contexts.

Beyond its contributions to the CABI framework, this study expands the scope of attachment theory by extending its application from interpersonal relationships to human-technology interactions. The findings provide a refined conceptual model that can serve as a foundation for future research on the psychological mechanisms underlying user-platform interdependence.

### 6.3 Practical implications

The findings of this study offer actionable insights for platform operators aiming to enhance user engagement and retention. Addressing cognitive factors like attraction, interactivity, and perceived enjoyment should be a primary focus. Platforms can achieve this by introducing customizable interfaces that allow users to personalize themes and layouts, thereby increasing their sense of involvement. Leveraging AI-driven recommendation systems can also improve perceived enjoyment by delivering tailored content that aligns with user preferences.

Emotional attachment can be cultivated by creating a sense of community and fostering meaningful user experiences. Furthermore, addressing privacy concerns, maintaining high-quality content, and implementing clear regulatory measures can reinforce trust and long-term loyalty (Yang L. et al., 2023).

Optimizing functional features is equally critical. Platforms should focus on usability enhancements, such as improving streaming quality, introducing advanced interactive tools, and refining content search functions.

## 7 Limitations and future research directions

While the study does not aim to directly integrate TAM, U&G, S-O-R, and Flow theories into a unified framework, it draws upon key constructs from these models—such as usability, hedonic motivation, environmental stimuli, and affective immersion—to inform the design of the CABI-based attachment mechanism. This approach allows for a more comprehensive understanding of users’ continuance intention on pan-entertainment live streaming platforms.

Nonetheless, several issues warrant caution. The sample is confined to domestic users, which may limit cross-cultural generalizability. The model also focuses on psychological pathways while omitting external forces such as AI-recommendation transparency, platform competition, and users’ social identity. These factors could mediate or moderate the attachment–continuance chain and may vary across markets.

Future work should test the model with multinational samples, incorporate technological and competitive variables, and employ longitudinal, multi-platform data to capture how market dynamics reshape user loyalty over time.

## 8 Conclusion

This study makes significant theoretical contributions to the understanding of user retention in digital platforms. By embedding emotional and functional attachment as mediators, the research challenges the traditional cognition-to-behavior pathway in the CABI framework (Liang and Kee, 2018). The findings suggest that affective responses, such as emotional attachment, and functional dependencies, such as usability and platform reliability, work together to translate cognitive evaluations (e.g., platform attraction, interactivity) into sustained behavioral intentions. This expanded perspective allows for a more nuanced analysis of user behavior in interactive environments.

Another key contribution lies in the contextualization of attachment theory within Pan-entertainment live streaming platforms. While previous studies have applied attachment theory to brand relationships (Boateng et al., 2020) and social media engagement (Wan et al., 2017), its relevance in live streaming environments has remained largely unexplored. This research demonstrates that users not only form emotional bonds with content creators but also develop attachments to the platform itself, shaped by features such as real-time interaction, tipping mechanisms, and personalized content delivery. These insights offer a new perspective on how attachment operates in digital media consumption.

The identification of a dual-path mediation mechanism further refines the understanding of attachment dynamics. The findings indicate that entertainment value enhances emotional attachment but does not significantly contribute to functional attachment, revealing a distinct divergence in how users experience and interact with Pan-entertainment live streaming platforms. This distinction is crucial for platform designers seeking to balance hedonic and utilitarian factors to improve long-term user retention.

This study uniquely integrates emotional and functional attachment as dual-path mediators in analyzing continuance intention on pan-entertainment platforms, thus enriching the theoretical dimensions of the CABI framework. However, the study’s focus on Chinese users may restrict the generalizability of its findings; future research should incorporate cross-cultural comparisons to validate and extend the proposed model. Expanding future research to include cross-cultural comparisons could offer deeper insights into how cultural differences shape attachment dynamics. Additionally, this study does not explore the role of emerging technologies such as AI-driven personalization and VR/AR integration in influencing attachment formation. Investigating these factors could further clarify the evolving nature of user retention mechanisms in live streaming ecosystems. Nonetheless, this research provides a foundational framework for understanding how emotional and functional attachment contribute to sustained engagement, offering theoretical and practical insights for the design and management of digital platforms.

## Data Availability

The original contributions presented in the study are included in the article/Supplementary material, further inquiries can be directed to the corresponding author.
