# Supplementary material for: Why do users stay? Emotional vs. functional attachment in pan-entertainment live streaming platforms
Source: Front Psychol. 2025 Jul 21;16:1623568. doi: 10.3389/fpsyg.2025.1623568 (PMC12320146; doi:10.3389/fpsyg.2025.1623568)
Supplement: Supplementary file 1 [file Table_1.docx]

| **Questionnaire content** | | |
| --- | --- | --- |
| Variant | Theme | Source |
| Attraction | I think the interface of the pan-entertainment live streaming platform has beautiful icon ideas and comfortable font size | 1.Sustainability of Live Video Streamer’s Strategies: Live Streaming Video Platform and Audience’s Social Capital in South Korea  2.Analysis of the Effect of Attraction, Expertise, Interaction, Image Satisfaction, and Advertising Trust on Purchase Intention in Social Media Influencer Marketing on Erigo Consumers in Indonesia |
|  | I think the interface of the pan-entertainment live streaming platform is vivid and colorful and can attract people's eyes |  |
|  | I think the interface of the pan-entertainment live streaming platform is layered and can be observed directly in the center of the screen |  |
|  | I think the pan-entertainment live platform interface has a unique visual presentation that fits the aesthetic |  |
| Perceived Enjoyment | I like to use the Pan-Entertainment Live Streaming Platform | 1.What drives live-stream usage intention? The perspectives of flow, entertainment, social interaction, and endorsement  2.Understanding perceived enjoyment and continuance intention in mobile games |
|  | I think it's a pleasure to use a pan-entertainment live streaming platform |  |
|  | My experience with the PanEntertainment Live platform was enjoyable! |  |
|  | I think it's pretty cool to use a pan-entertainment live streaming platform |  |
| Interactivity | I often interact with people on live pan-entertainment platforms | 1.Knowledge Sharing Platforms: An Empirical Study of the Factors Affecting Continued Use Intention  2.Sustainable customer retention through social media marketing activities using hybrid SEM-neural network approach |
|  | Feeling closer to the anchor when using a pan-entertainment live streaming platform |  |
|  | When I use a pan-entertainment live streaming platform, I can easily exchange and share opinions with the anchor or other viewers |  |
|  | When I watch live streams using the PanEntertainment live streaming platform, the anchors provide ample opportunities to answer and ask questions |  |
| Entertainment | Exciting program content for a pan-entertainment live streaming platform | 1.What drives live-stream usage intention? The perspectives of flow, entertainment, social interaction, and endorsement  2.Uses and Gratifications Theory and E-Consumer Behaviors: A Structural Equation Modeling Study  3.The Impact of TikTok User Satisfaction on Continuous Intention to Use the Application |
|  | I'm stimulated by the pan-entertainment live streaming platform's programs |  |
|  | I was entertained by the pan-entertainment livestreaming platform's program |  |
|  | The pan-entertainment live streaming platform has programs that I find attractive |  |
| Emotional Attachment | I'd like to be friends with other users of the PanEntertainment Live platform | 1.An exploration of the role played by attachment factors in the formation of social media addiction from a cognition-affect-conation perspective  2.Research on the Influencing Factors of Users' Continued Usage Intention of Mobile Reading Apps from the Perspectives of User Experience and Status Bias |
|  | Live pan-entertainment platforms are important to me |  |
|  | I want to interact with its users after using a pan-entertainment live streaming platform |  |
|  | I use a pan-entertainment live streaming platform that is more in line with my lifestyle and personality |  |
| Functional Attachment | In order to accomplish my experience or other goals, pan-entertainment live streaming platforms can provide data resources | 1.Indifference Dependence and Immersion Experience: A Study on Mobile Short Video Users' Continued Usage Intention. Library and Information Work  2.How attachment affects users’ continued use intention of tourism mobile platform: A user experience perspective |
|  | In order to accomplish my experience or other goals, a pan-entertainment live streaming platform can provide technical support |  |
|  | In order to accomplish my experience or other goals, the pan-entertainment live streaming platform is able to update the information push in a timely manner |  |
|  | My account's personal information is protected during my use of the PanEntertainment Live Streaming Platform |  |
| Continuance Intention | The pan-entertainment live streaming platform is worth my continued viewing pleasure | 1.Mobile-lizing continuance intention with the mobile expectation-confirmation model: An SEM-ANN-NCA approach  2. Investigating Health and Fitness App Users’ Stickiness, WOM, and Continuance Intention Using S-O-R Model: The Moderating Role of Health Consciousness |
|  | The live pan-entertainment platform deserves my continued advocacy |  |
|  | I will continue to use the PanEntertainment live streaming platform to watch live streams in the future |  |
|  | I would recommend others to use the PanEntertainment live streaming platform to watch live streams |  |
